# Assessing Potential Spawning and Nursery Habitat Availability in the River Rhine for the Critically Endangered European Sturgeon

**DOI:** 10.1002/aqc.70016

**Published:** 2024-11-25

**Authors:** Niels W. P. Brevé, Debora A. J. van Dieren, Marc Weeber, Erik Mosselman, Leopold A. J. Nagelkerke, AlberTinka J. Murk, Anthonie D. Buijse

**Affiliations:** ^1^ Aquaculture and Fisheries Group Wageningen University Wageningen the Netherlands; ^2^ Marine Animal Ecology Group Wageningen University Wageningen the Netherlands; ^3^ Sportvisserij Nederland Royal Dutch Angling Alliance Bilthoven the Netherlands; ^4^ Department of Freshwater Ecology and Water Quality Deltares Delft the Netherlands; ^5^ Faculty of Civil Engineering and Geosciences Delft University of Technology Delft the Netherlands

**Keywords:** *Acipenser sturio*, coastal infrastructure, damming, habitat suitability modelling, inland navigation, regulated river

## Abstract

Information about reproductive habitat and migration pathways is of paramount importance to restore migratory fish species. This study assesses the availability of spawning and nursery habitats for the European sturgeon ( *Acipenser sturio* ) in the delta and lower Rhine (covering over 350 river kilometres) as part of a larger feasibility assessment for a future restoration of this critically endangered species. The general approach has three steps: (1) the identification of the species' specific habitat requirements, based on a systematic literature review; (2) the collection and preprocessing of data from two countries, including the 1D and 2D modelling of water depths and flow velocities; and (3) GIS‐based mapping of spawning and nursery habitat. Based on a HSI score of 1, we identify a total of 0.75 km^2^ as minimal spawning habitat, potentially suitable for approximately 2500 female European sturgeons (one spawning site would use ~300 m^2^). This is sufficient, as currently, only an estimated maximum number of 750 adults exist. Suitable spawning habitat is mainly located in the German state of North Rhine‐Westphalia, whereas suitable nursery habitat is mainly located in the Netherlands. The availability is, however, significantly reduced by coastal infrastructure (damming) and inland navigation. The insights gained can be used to assess the current suitability of the river Rhine for the species' reintroduction and to identify opportunities for habitat restoration and protection for various life stages. The outcomes thus play an essential role in the conservation of the species. In addition, the modelling approach developed could be applied to other northwestern European rivers. This broader application would allow intercomparison and support decisions about which rivers are best suited for future reintroduction of the critically endangered European sturgeon.

## 1 Introduction

Historically, the anadromous European sturgeon ( *Acipenser sturio* ) occurred in all marine and large river basins of northwestern Europe (Williot et al. 2002). Around 1850, the species' populations began to decline due to various human activities, in particular overfishing, damming, canalisation and environmental pollution (Debus 1997; Holčík et al. 1989; Williot et al. 2002). In the course of the twentieth century, the European sturgeon became extirpated across its entire former range, but for one small relict population in the Gironde river basin, which naturally reproduced at this site until 1994 (Williot and Castelnaud 2011). The species was only saved from extinction by capturing the last individuals from the Gironde river basin, which formed the basis of a programme of artificial reproduction (Williot and Castelnaud 2011; Williot et al. 2002).

Today, the European sturgeon is listed as critically endangered by the IUCN (Gessner et al. 2022) and still depends for its survival on ex situ rearing and the release of offspring (stocking) (France Ministère de l'Écologie 2020). Unfortunately, no natural reproduction has been observed yet in the Gironde river basin or elsewhere in the world, notwithstanding the in situ habitat restoration performed (Gessner et al. 2022). To improve the species chances of return, it is paramount to develop further conservation activities. In the 1990s, a second ex situ broodstock in Berlin had already been established for reintroduction of the species to the river Elbe (Gessner et al. 2011). Since 2009, the river Rhine has been under consideration for a potential third reintroduction (de Nie and Ommering 1998), whereas in 2020, a ‘First Action Plan for the European sturgeon ( *A. sturio* ) for the Lower Rhine’ has been developed (Visser et al. 2020). This has been the result of several literature and field studies, including experimental releases of young European sturgeons in 2012, 2015 (Brevé et al. 2019) and recently also in 2023 and 2024. The conservation activity for the Rhine has thus been underway for 15 years. However, before a decision can be made to start a full reintroduction programme, a thorough feasibility assessment is required to prevent the waste of time, money and extremely rare fish, on a potentially failing reintroduction.

The Rhine, a former stronghold of the European sturgeon (Brevé et al. 2022; Kinzelbach 1987) has certain advantages for restoring a population of its largest fish species. The Rhine is one of only a few large European rivers that still has an open connection between the North Sea, the port of Rotterdam and the Rhine's middle and upper reaches across 850 river kilometres (Puijenbroek et al. 2019; Uehlinger et al. 2009). The Rhine has been subjected to major restoration efforts, cleaning up its historical industrial pollution (albeit not everywhere in the river bed) (Dieperink 1998; Mostert 2009; van Dijk, Marteijn, and Schulte‐Wülwer‐Leidig 1995; Villamayor‐Tomas et al. 2014), and following the objectives as set out in the European Water Framework Directive and the Habitats Directive (EEC 1992; European Union 2000; ICPR 2018), the Rhine has seen a strong improvement of its water quality and ecological status (Plum and Schulte‐Wülwer‐Leidig 2014). The latter is underlined by the partial recovery of several of the Rhine's migratory fish populations, such as river Lamprey ( *Lampetra fluviatilis* ) and Houting ( *Coregonus oxyrinchus* ), and there are initial signs of recovery of Allis shad ( *Alosa alosa* ) (Brevé et al. 2014; Hundt et al. 2015; ICPR 2018).

However, the Rhine also has several disadvantages for reintroducing its extinct migratory fish species. A significant part of its delta is obstructed by dikes and storm surge barriers that impair the fish migration routes (Bij de Vaate et al. 2003; Breukelaar et al. 2009; Brevé, Vis, and Breukelaar 2019; Brevé et al. 2013; Verbiest et al. 2012). The Rhine is also one of the most intensively navigated rivers in the world (Mako and Galieriková 2021; Uehlinger et al. 2009), and shipping may induce harmful water movements and mechanically damage fish through ship propeller strikes (Brevé et al. 2019; Spierts 2016). As such, not only some remaining environmental pollution in the river bed also damming and shipping are still pressures of sufficient concern to be included in the feasibility assessment.

The feasibility assessment thus aims to estimate the impact of these disadvantages and if necessary design management activities to mitigate these, as is outlined in the Rhine sturgeon action plan (Visser et al. 2020). Unfortunately, the feasibility assessment lacks vital information about the availability of the species' spawning and nursery grounds. As the Rhine's sturgeon population became already virtually extinct in the 1920s, at a time when fisheries biologists were unaware of the species' complex life‐cycle, no historical information was collected of the spawning and nursery grounds (Hoek 1910; Verhey 1963; Verhey 1949). However, we propose that the collection of historical data on sturgeon catches will help to identify the historical key habitats of the sturgeon, whereas the collection of data from the current rearing of fertilized eggs and yolk sac hatchlings will help to identify the required parameters for spawning and nursery. Modelling of the Rhine for these parameters will then identify suitable spawning and nursery sites. The present study therefore aims to address an important part of the feasibility assessment, by answering the question: Where and how much suitable spawning and nursery habitats of the European sturgeon can be found in the Rhine? Answering this question will support a science‐based go/no‐go decision for reintroduction of the species into this large, heavily modified European river.

## 2 Materials and Methods

### 2.1 Study Area

**FIGURE 1:**
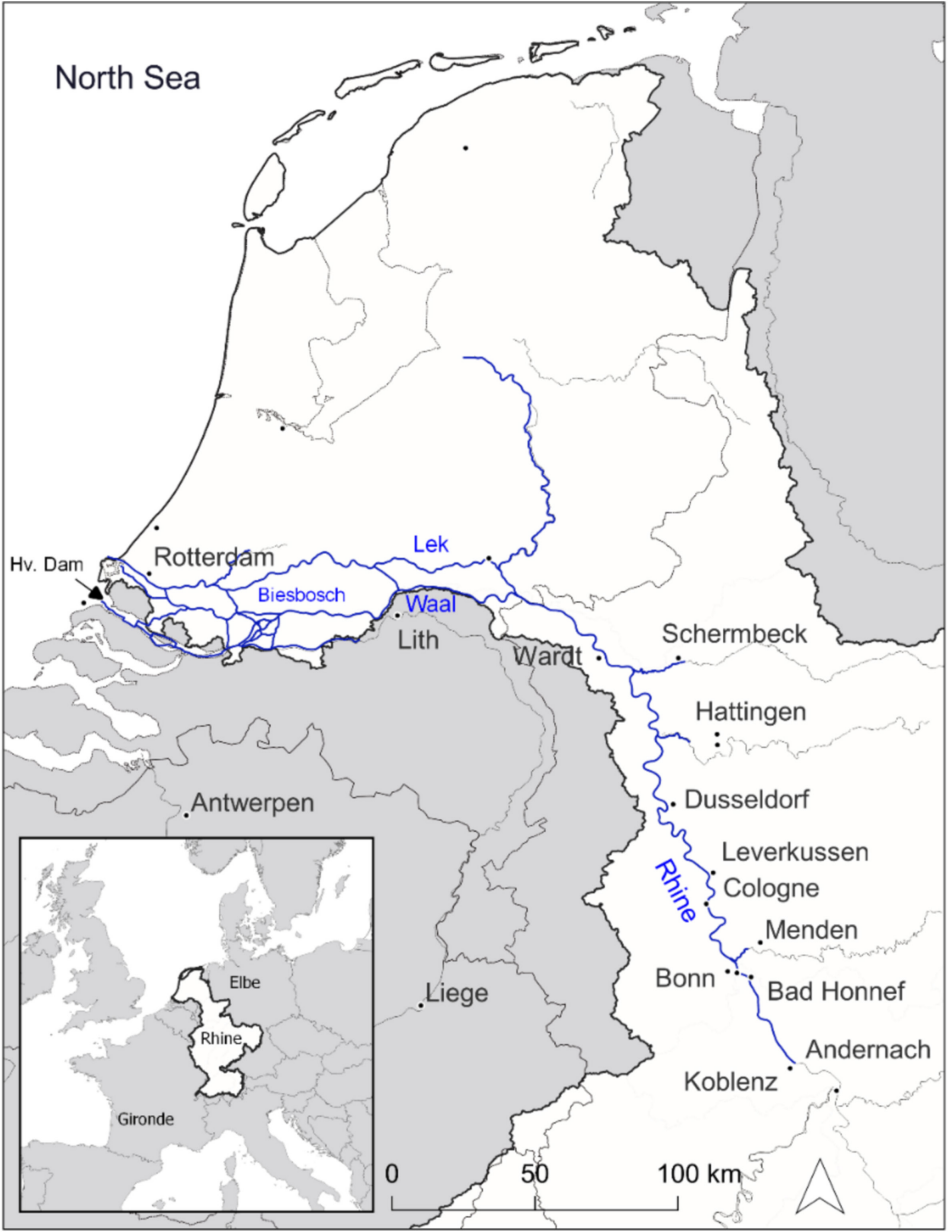
Map overview of the study area. In white: the delta and lower part of the Rhine river basin. In blue: three combined SOBEK models (see Table 1), whereby modelling of the Rhine was done for the Netherlands and the German state of North Rhine‐Westphalia (from the North Sea until Bad Honnef). Hv. Dam = Haringvliet Dam.

The study area is based on the European sturgeon's historical spatiotemporal occurrences of capture reports of sturgeons, as described in Brevé et al. (2022). From this study, it was deduced that adult sturgeon on their spawning run occurred in the Rhine from the end of April to mid‐August, with a clear peak during the last week of June. Approximately 95% of the historical sturgeon catches were collected from the delta of the rivers Rhine and Meuse in the Netherlands and from the Rhine's main stem within 250 river km from the sea (Brevé et al. 2022). To include most of the catches, presumably aligning the localisation of the historical spawning sites, the study area comprises the Rhine in the Netherlands and the German state of North Rhein‐Westphalia (NRW), that is, from the port of Rotterdam to Bad Honnef (a town near Bonn, see Figure 1). For comparison: in the Gironde river basin, European sturgeon also spawned between May and August, whereby these spawning grounds were mainly located upstream of the tidal zone between river km 170 and 245. For the Dordogne sturgeon, spawning sites were found between Pessac and Bergerac and for the Garonne between Meilhan and Agen (Bismuth and Lauronce 2019; Jego et al. 2002).

### 2.2 General Approach

The general approach consists of three main steps: (1) a systematic literature review of historical catches and current data from sturgeon rearing practices to identify the key abiotic parameters for European sturgeon spawning and nursery; (2) the collection of data from the study area and preprocessing of water depths and flow velocities using 1D and 2D modelling techniques; and (3) GIS‐based mapping of spawning and nursery grounds for European sturgeon in the Rhine. Noticeably, in Section 4, we evaluate the outcomes and discuss the potential negative effects of damming and shipping, as these may reduce the suitability and hamper the sturgeon migration pathways to and from the spawning and nursery habitats.

### 2.3 Step 1: Systematic Literature Review

The systematic literature review was conducted through the platforms of the Natural Science Collection ProQuest (https://www.proquest.com/products‐services/natural_science.html) and Scopus (www.elsevier.com/solutions/scopus). The following *query string* was applied in ‘advanced search’ in titles, abstracts and summaries (* = wildcard): (‘ *Acipenser sturio* ’ *or* ‘ *Acipenser oxyrinchus* ’) *and* (egg *or* yolk *or* embryo *or* larv* *or* depth *or* discharge *or* juvenile *or* oxygen *or* O^2^ *or* pH *or* nursery *or* salinity *or* spawn* *or* substrate *or* sediment *or* temperature *or* tide *or* tidal *or* turbidity *or* sediment *or* velocity *or* current). After eliminating duplicate information, scanning all abstracts and further snowballing (citation tracking), in total, 142 documents (articles, book chapters, reports and theses) remained that were of interest to this study (for references, see Supporting Information S1).

### 2.4 Step 2: Data Collection and Modelling (Preprocessing)

The identified ranges of values for key abiotic parameters for European sturgeon spawning and nursery habitats can be divided into (1) parameters that hardly change during one spawning season: river morphology (digital elevation model, DEM) and substrate composition (sediment type, grain size and sorting), and (2) parameters that fluctuate on a daily basis in a highly dynamic riverine environment: river discharge, water depth, flow velocity, water temperature, oxygen saturation and pH. Most data were obtained from several Dutch and German governmental agencies (see Table 1). However, water depths and flow velocities were derived from a hydrodynamic model because these data are not measured on a scale with enough detail to enable the identification of potential spawning and nursery sites.

**TABLE 1:** Data collection.

Data type (unit)	The Netherlands	North Rhine‐Westphalia, Germany
Digital Elevation Model (DEM), 1 × 1 m^2^ resolution grid cells derived from DEM and multibeam sonar bathymetric survey data.	RWS Baseline https://iplo.nl/thema/water/applicaties‐modellen/watermanagementmodellen/baseline/	WSV; Digitales Geländemodell, 1m: https://www.opengeodata.nrw.de/produkte/geobasis/hm/dgm1_xyz/dgm1_xyz/
Sediment grain size (diameter) in D10, D50, and D90 for every 500 m, albeit with some data gaps (largest = 11 km).	RWS; Fugro (2002) https://data.4tu.nl/datasets/c6e7c1ff‐44e6‐46f9‐9b30‐e010e91a97dd	Staas (2017); BafG https://geoportal.bafg.de/ggina‐portal/
Rhine water level (m above NAP)	RWS Waterinfo https://waterinfo.rws.nl/#/nav/bulkdownload	WSV https://www.pegelonline.wsv.de/gast/start
River discharge (m^3^/s)		BafG https://portal.grdc.bafg.de/applications/public.html?publicuser=PublicUser#dataDownload/Stations
Water temperature (°C), O^2^ saturation (%), pH		LANUV https://www.opengeodata.nrw.de/produkte/umwelt_klima/wasser/oberflaechengewaesser/gues/
SOBEK models	Deltares, reference websites for the models:sobek‐rmm‐vzm‐j15_5‐v4 (Rhine‐Meuse Estuary, https://iplo.nl/thema/water/applicaties‐modellen/modelschematisaties/zuidwestelijke‐delta/), sobek‐rijn‐j22_6‐v1a1_rwsos (NL Rhine branches), and sobek‐rijn‐j17_5‐v2‐rwsos_merge (DE Rhine to Andernach): https://iplo.nl/thema/water/applicaties‐modellen/modelschematisaties/rivieren/ sobek‐rijn‐j22_6‐v1a1_rwsos (NL Rhine branches), and sobek‐rijn‐j17_5‐v2‐rwsos_merge (DE Rhine to Andernach).	
Water depths (m) and Flow velocities (m/s)	Obtained from hydrodynamic modelling, using SOBEK.	

*Note:* Data sources: For the Netherlands, the data were obtained from Rijkswaterstaat, the executive agency of the Ministry for Infrastructure and Water Management (RWS) and Deltares. For the German state of North Rhine‐Westphalia, the data were obtained from the Bundesanstalt für Gewässerkunde (BafG), Wasserstraßen und Schifffahrtsverwaltung des Bundes (WSV), Das Landesamt für Natur, Umwelt und Verbraucherschutz (LANUV), Wasser‐ und Schifffahrtsamt Duisburg‐Rhein (WSA), and Landesamt für Natur, Umwelt und Verbraucherschutz Nordrhein‐Westfalen (LANUV).

Among existing models, only 1D models covered the full study area from the port of Rotterdam in the Netherlands to Bad Honnef in Germany. We therefore merged three SOBEK models into a single hydrodynamic model that covered the full study area (see Figure 1). SOBEK's partial models (for references, see Table 1) simulate complex flows and water‐related processes, with hydrodynamic 1D and 2D simulation and robust computational methods at any scale.

We used the resulting SOBEK model to calculate water levels along the Rhine river basin for discharges in June–August according to a dry, mean and wet scenario: P10, P50 and P90 respectively, whereby 10, 50 and 90 are percentile occurrences. The corresponding main inflow river discharges to the SOBEK model were 23, 35 and 73 m^3^/s at Hattingen; 10, 21 and 49 m^3^/s at Menden; 1301, 1976 and 2499 m^3^/s at Andernach; 20, 23 and 36 m^3^/s at Schermbeck; and 82, 135 and 245 m^3^/s at Lith (for locations see Figure 1). These discharges were derived from time series for years 1964–2020.

The 1D outputs for water levels were translated into 2D water depths by using the bed levels of a DEM that cover the area between the winter dikes (see Table 1, Netherlands (NL): RWS Baseline; Germany (DE): WSV Digitales Geländemodell). The water depth $d_{k}$ was calculated for each grid cell k by subtracting the local bed level $z_{k}$ from the water level h at the nearest SOBEK *observation point* (river kilometre): $d_{k}=h-z_{k}$. Flow velocities were calculated for the *reaches* (trajectories) between consecutive observation points, by deriving water depths from water levels at these observation points and bed levels according to the DEM and representative SOBEK cross‐sections for the reaches. We calculated depth for observation points and flow velocities for reaches. We divided each cross‐section into 150 bins j (vertical strips) with equal width ΔB. Applying the Chézy (1776) equation and the definition of discharge, the flow velocity at each bin was calculated under the assumption of steady uniform flow by

$$u_{j}=\frac{Q}{\mathrm{\Delta B}}\frac{d_{j}^{1/2}}{\left(d_{1}^{3/2}+d_{2}^{3/2}+..+d_{150}^{3/2}\right)},$$

where Q denotes the total discharge through a cross‐section. We compared initial results with those from a 2D hydrodynamic model of the Dutch Rhine branches (dflowfm2d‐rijn‐j22_6‐v1a; Kosters and Visser 2022) for a 10 km long test area. This revealed that the 1D results *overestimated* flow velocities in shallow areas (<4 m) and *underestimated* flow velocities in deep areas (>4 m). We used this to introduce a depth‐dependent correction factor in our method. Furthermore, the formula *overestimated* flow velocities in deep areas outside the main channel, such as lakes and harbours, where virtually no water flows in the months June–August (dry summer months). Grid sells in such areas were therefore set to a low flow velocity of 0.1 m/s.

Sediment grain size was superimposed on the DEM grid based on observational data. We used sediment grain size data (grain diameter; see Table 2) from measurements to determine the minimum, medium and maximum D_50_ grain size. Despite slight coarsening of riverbed sediment on a scale of centuries (Frings 2011), the main picture of a gravel bed in the German Niederrhein and a sand‐gravel bed in the Dutch Rhine branches has remained present over millennia (Berendsen and Stouthamer 2001; Havinga 1993). For the Netherlands, these grain sizes are available for every kilometre, on the left, in the centre and on the right side of river. For Germany, only one set of grain sizes is available per cross‐section, every 500 m, with gaps up to a river stretch of 11 km. Data on sediment grain size were not measured in the estuary of the Rhine and Meuse rivers in the Netherlands but were assumed to be approximately 1 mm based on Fugro (2002).

**TABLE 2:** Parameter‐setting for GIS‐based mapping of spawning and nursery habitat for European sturgeon ( *Acipenser sturio* ). This is a summary of data from the literature review. For the full dataset and the references, see Supporting Information S1.

Habitat		Spawning and hatching	Nursery
**Life‐stage**		**Fertilized‐eggs & yolk‐sac larvae**	**Larvae (< 50 mm) & juveniles 0** ^**+**^ **(< 300 mm)**
**Location in river basin**		River main stem, mainly within 250 river km, ~80–100 km upstream of the tidal zone.	River main stem, lower reaches, and estuary.
**Main season**		June–August	June–March
**Total length**		Yolk‐sac larvae ~15 mm	15–300 mm
**Age**		Hatching after 3–7 days	0–11 months old
**Parameter**	**Classification (category)**	**Parameter‐setting for modelling**	
**Water depth**	0–2 m (shallow)	0.25	0.75
	2–4 m (moderately deep)	1	1
	4–6 m (deep)	0.75	1
	> 6 m (very deep)	0.5	0.75
**Flow velocity**	0–0.5 m/s	0.5	1
	0.5–1.0 m/s	1	0.5
	1.0–1.5 m/s	0.75	0.25
	1.5–2.0 m/s	0.5	0
	> 2.0 m/s	0	0
**Grain Ø (substrate composition in ASTM grid D50)** Eggs will not be deposited in areas rich in silt and clay.	< 0.075 mm (silt and mud)	0	1
	0.075–2 mm (sand)	0	0.75
	2–16 mm (gravel fine)	0	0.25
	16–31.5 mm (gravel medium)	1	0
	> 31.5 mm (gravel and large rocks)	0.75	0
**Water temperature** Embryonic survival peaks at 20°C. Larvae upper tolerance limit is between 26 and 30°C, and lower tolerance limit < 12°C.	< 15°C	0	0.5
	15°C–17°C	0.25	0.75
	17°C–19°C	0.75	1
	19°C–21°C	1	1
	21°C–23°C	0.5	0.5
	> 23°C	0.25	0
**O** ^**2**^ **saturation** Oxygen depletion induces sublethal effects at 70% O2 saturation and lethal effects at 50% O2 saturation.	< 50%	0	0
	50–70%	0	0.5
	70–90%	0.5	0.75
	> 90%	1	1
**pH** Minimum 5.5 Median 7.6 Maximum 8.7	< 5	0	0
	5–6	0	0
	6–7	0.5	0.5
	7–8	1	1
	8–9	0.5	0.5
	> 9	0	0

### 2.5 Step 3: GIS‐Based Mapping of Spawning and Nursery Habitats

Using the Habitat Evaluation Procedure (HEP) (US Fish and Wildlife Service 1980), we mapped the Habitat Suitability Index (HSI) and the limiting variables for spawning and nursery areas for European sturgeon (HSI maps). These HSI maps were created by confronting the derived species' criteria (see Table 2) with the modelled values of water depth, flow velocity and sediment grain size (sedmax, sedmed and sedmin) for the Rhine, for three scenarios (P10, P50 and P90). We then estimated the total sum of suitable spawning and nursery sites for the river Rhine in km^2^ for these criteria and scenarios (see Table 3).

**TABLE 3:** Available area (in km^2^) of potentially suitable habitat for spawning and nursery of European sturgeon ( *Acipenser sturio* ) in the river Rhine. The data are obtained through modelling of the ranges of water depth, flow velocity, sediment grain size, and overall (combined). See Table 2 and Supporting information S1 for the parameter settings. HSI scenario's use water level (P10, P50 and P90) and sediment grain size. Notably, we use sedmed and sedmax for spawning sites as mature European sturgeons prefer coarse substrates and sedmed and sedmin for nursery sites as juvenile European sturgeons prefer finer grain sizes. Colour indications range from red to green and indicate the quality from unsuitable to suitable.

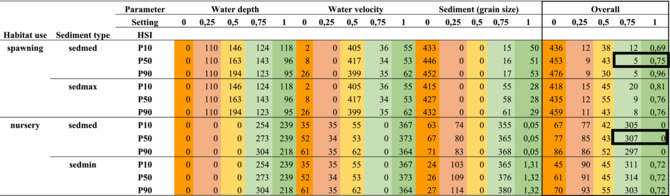

For this method, we chose to apply predetermined suitability categories and based the suitability value on the minimum value methodology (Zhang et al. 2016), resulting in a hard cut‐off between classes instead of a gradual fuzzy logic approach. We used D‐Eco Impact (Weeber et al. 2024), to map spawning and nursery areas within the study area, based on the modelled values of parameters water depth, flow velocity and sediment grain size.

The results of the modelled values in addition to the suitability mapping exercise were presented in QGIS (Version 3.28.2). For more information, about our modelling approach, see Supporting Information S2.

## 3 Results

### 3.1 Abiotic Parameters That Identify Spawning and Nursery Habitats

**FIGURE 2:**
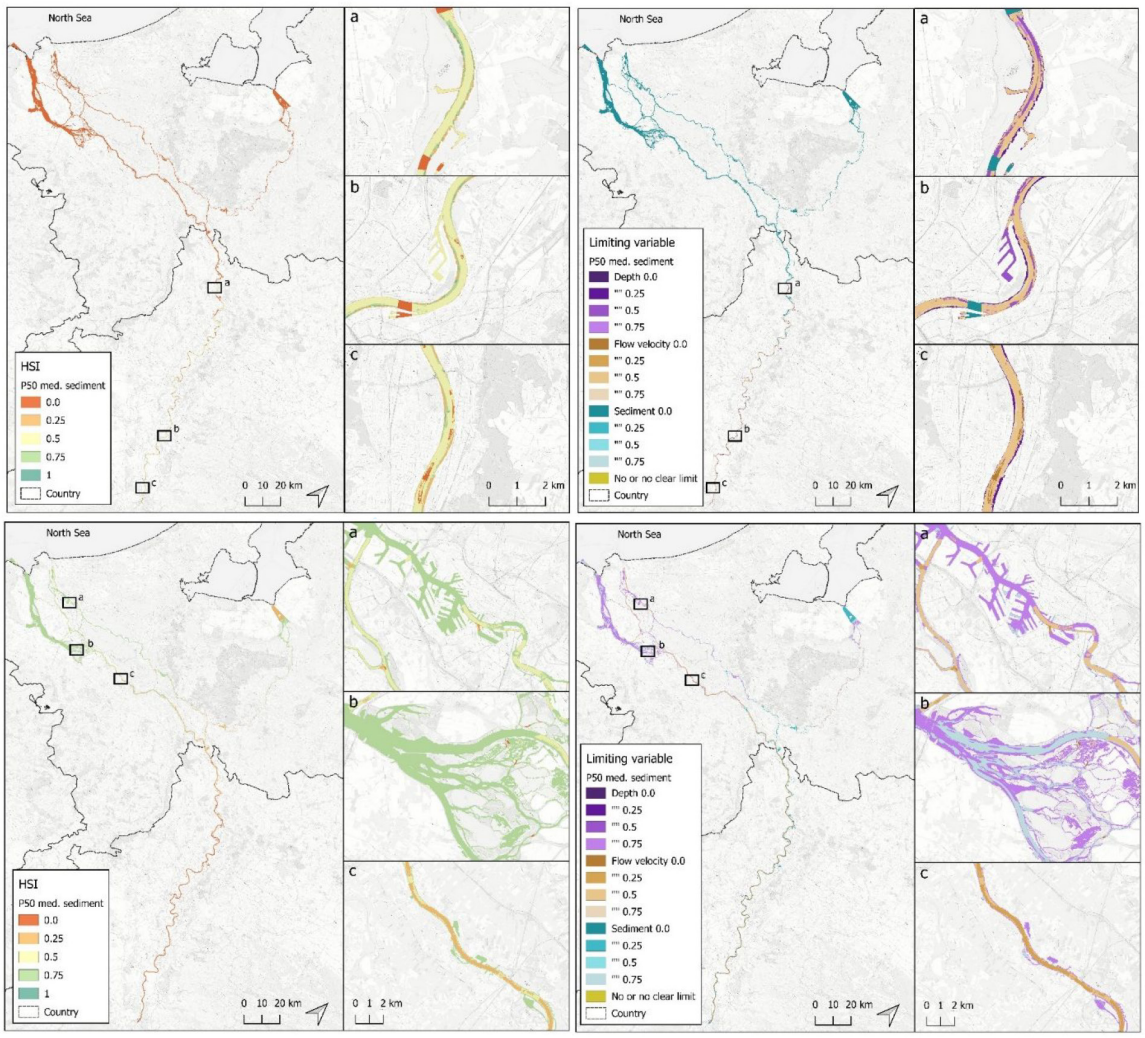
Potential areas in the river Rhine for European sturgeon *spawning* (TOP) and *nursery* (BOTTOM), obtaind through GIS‐based mapping. Inlay maps (a–c) show examples in more detail. Names are those of towns, for example, Wardt and Bonn (for locations, also see Figure 1). Panels LEFT show sites that are *suitable* (habitat suitability index determined for scenario P50, medium sediment suitability) and panels RIGHT show sites that are *unsuitable* (limiting variables). TOP: a = Wardt (effect of sediment cut‐off),; b = Cologne (highly suitable); c = Bonn (effect of modelling/changes in flow velocity through calibration). BOTTOM: a = the port of Rotterdam (suitable; however, sediments are not fine enough); b = Biesboch freshwater delta (idem as a); c = the river branch Waal.

The systematic literature review identified the ranges (i.e., minimum, optimum and maximum values) of key habitat abiotic parameters that identify spawning and nursery habitats for the European sturgeon (see Supporting Information S1). The HSI parameter setting for modelling is scaled along a simple gradient ranging from unsuitable to suitable, with values 0, 0.25, 0.50 and 1 (see Table 2) estimated based on the literature review. Consequently, in our HSI modelling, the suitable reproductive habitats are limited by parameter values lower than 1 (see Figure 2).

### 3.2 Mapping Spawning and Nursery Habitats, Using HSI Scores

GIS‐based mapping using HSI scores revealed that the main suitable spawning and nursery sites in the Rhine are rougly divided over the two countries:spawning in NRW‐Germany and nursery in the Dutch Delta. Examples can be seen in Figure 2, where the top and bottom panels show spawning and nursery areas, respectively, and the left and right panels show suitable and unsuitable areas, respectively. The currently suitable *spawning* sites in the Rhine are mainly located in NRW‐Germany because of the high scores (> 0.75) for water depth, flow velocity and grain diameter. Unsuitable spawning sites in this area are limited by water depth (too shallow), flow velocity (too low) and partially grain size (too fine). The currently suitable *nursery* sites are mainly located in the Netherlands. In NRW‐Germany, the availability of suitable nursery sites is mainly limited by flow velocity (too high). For the Dutch Delta, the suitability of spawning habitat is mainly limited by grain size (too fine), whereas some limitations are caused by combined qualities of flow velocity, water depth and grain size. Notably, an HSI score of 1 for nursery sites was never obtained because the average and minimum of grain size do not reach the required P50 minimum value of 0.075 mm (see Table 2).

### 3.3 Availability of Spawning and Nursery Habitat for European Sturgeon in the Rhine

Based on a HSI overall score of 1 and sedmed P50, the Rhine comprises 0.75 km^2^ habitat suitable for spawning and 0 km^2^ suitable for nursery. The latter is because of the strict limitations set for sediment grain size (< 0.075 mm, see Table 2). As a result, hardly any location in the Rhine scored an overall HSI of 1 for spawning, and none for nursery (see Table 3). The choice of the parameter values is based on the systematic literature review, and possibly, our settings were too strict. It cannot be excluded, however, that currently no suitable nursery habitat is available. When for the HSI, an overall score of 0.75 and sedmed P50 are accepted, the Rhine provides 5.75 km^2^ (5 + 0.75) for spawning (mainly located in NRW Germany) and 307 km^2^ (307 + 0) as nursery habitat area (mainly located in the Netherlands) (see Table 3, indicated with the two black frames).

So how many female European sturgeons could probably spawn in the Rhine? According to Gessner and Bartel (2000), the carrying capacity of European sturgeon spawning grounds can be estimated based on the density of incubation (Derzhavin 1947). With an average fertility of one million eggs and an optimum density of 1000–3500 eggs/m^2^, the average spawning site size for one female would be approximately 300 m^2^ (Jakob 1996). Therefore, 0.75 km^2^ of suitable spawning sites would provide 2500 female European sturgeons with suitable spawning habitat (see Table 4).

**TABLE 4:** Number of female European sturgeons that could theoretically be provided with suitable spawning habitat based on a 300 m^2^ requirement. This is based on presence of area with certain characteristics; no other limiting factors were taken into account. HSI 0 = unsuitable, HSI 1 = optimal.

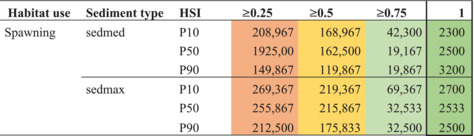

## 4 Discussion

### 4.1 Limitations

As the European sturgeon has been extinct in the Rhine since 1953 (Verhey 1963) and world‐wide European sturgeon has not spawned since 1994, the validation of the spawning sites by monitoring the habitat using live fish is impossible. The assessment had thus to be estimated from a set of parameters as compiled from the systematic literature review, while the study also encountered some data gaps and a lack of a 2D model for NRW‐Germany. Still, the literature review uses high quality information from current ex situ rearing plus information from historical and recent assessments of spawning sites, the latter especially from the Gironde river basin (Bismuth and Lauronce 2019; Guerri and Pustelnik 1996; Jego et al. 2002). Moreover, data gaps were marginal as measures of the Rhine are frequently updated by the Dutch and German governments (see Table 1), and our 1D modelling was detailed per river kilometre across the study area. Therefore, the parameter values, data and models used in this assessment to identify spawning and nursery habitats are to be considered of a good quality to answer the research question.

Based on the data and models, we have shown that the Rhine comprises sufficient habitat for approximately 2500 to 19,000 female European sturgeons to spawn, depending on a HSI of 1.0 or 0.75, respectively. This would be sufficient for the estimated maximum of 750 (sub)adult European sturgeons alive in the wild (Gessner et al. 2022). Nevertheless, it is important to also consider effects of the assessment that could imply either an overestimation or underestimation of habitat suitability.

### 4.2 Underestimation in the Modelling

There are two main reasons why the modelling may represent an *underestimation* of habitat suitability.

First, the study uses average parameter values and hard cut‐off values for parameter settings (Table 2), but European sturgeon is naturally capable of coping with a variety of environmental conditions during migrations upstream and downstream (Staaks, Kirschbaum, and Williot 1999). The real spatial variation in flow velocities is larger than the values from the 1D model in the GIS maps, and the water depths in the model are approximately 20 cm larger than the measured values due to the conversion from a 1D to a 2D model, which could be improved by calculating more observation points in the SOBEK model through linear interpolation between existing observation points. Therefore, suitable habitat may have been left out the assessment due to our modelling approach.

Second, there may be additional suitable habitat for sturgeons to spawn outside the study area, located in the Middle and Upper Rhine between Bonn and Iffezheim.

### 4.3 Overestimation in the Modelling

In contrast, there are three main reasons why the modelling may represent an *overestimation* of habitat suitability that are related to (1) a neglect in the assessment of the effects of temperature, oxygen saturation and pH that can be related to climate change and some remaining environmental pollution, and (2) the physical influence of damming and (3) shipping.

First, adult European sturgeons are late‐spring and summer spawners (main season: May–August). They can cope with various microclimates during their migrations (Staaks, Kirschbaum, and Williot 1999) and are tolerant to oxygen deficiencies (Holčík 1989). However, the youngest life stages of fertilized eggs and yolk sac larvae are sensitive to aberrant temperatures and O_2_ saturation and to a lesser extent to variations in pH (Delage et al. 2014; Delage et al. 2019; Delage, Rochard, and Cachot 2015). Oxygen levels below 6 mg/L are harmful for embryos (van Winden et al. 2000), whereas pH levels are optimal between a pH of 7 to 7.8 and should not go below 5.5 or above 8.7 (Delage et al. 2019; Williot et al. 2009). Thanks to improved water quality, the Rhine's O_2_ levels were already at safe levels in 2009 at approximately 9–10 mg/L (Uehlinger et al. 2009), whereas the mean annual pH (1995–2004) varies between 7.9 and 8.3 and decreases downstream (Uehlinger et al. 2009). Therefore, oxygen saturation and pH in the Rhine do not seem to be a problem for the reintroduction of European sturgeon. For water temperature, however, there are reasons of concern. Embryonic survival is highest at 20°C with a range of 17°C°C–22°C, whereas the upper tolerance limit is between 26°C and 30°C (Delage et al. 2019). The Rhine water temperatures are currently still in the normal temperature range during summer months. However, due to anthropogenic thermal effluents and climate change, the measured summer water temperature at Koblenz increased by over 2°C between 1978 and 2011 and the number of days per year with water temperatures above 22°C increased as well (ICPR 2013). Future projections show that Rhine water temperatures will further increase (Hardenbicker et al. 2017; Lassalle et al. 2010; Lassalle and Rochard 2009). Therefore, rising water temperature, although not an immediate threat, could become a problem in the long term.

Second, although the main stem of the Rhine is open between the port of Rotterdam and the first hydropower plant at Iffezheim at river km 334, the Haringvliet Dam (see Figure 1) between the North Sea and the historical estuary of the rivers Rhine and Meuse forms a major barrier to fish migration (Bij de Vaate et al. 2003; Breukelaar 2017; Brevé, Vis, and Breukelaar 2019; Brevé et al. 2019). The dam is open for high river discharges to the sea, and it was envisaged to also enable fish migration through a small opening at low river discharges. However, the opening has remained limited (even absent during dry summer months when the sluices are closed) to prevent salinization of the freshwater reservoir (Noordhuis 2017). This hampers safe and accessible migration to and from habitats. The latter was underlined by the experimentally released young European sturgeon, which on their downstream migration largely avoided the Haringvliet route altogether (Brevé et al. 2019). Fish migrated into the North Sea via the port of Rotterdam that carries much more river discharge (Brevé et al. 2019). These findings show that damming of the former estuary of the rivers Rhine and Meuse results in fish omitting potential suitable nursery habitat.

Third, and last, the Rhine is one of the most intensively navigated rivers in the world (Mako and Galieriková 2021). The adult European sturgeons prefer to swim in the deep and fast flowing river sections of Europe's largest rivers and may use the river's scour holes as resting and hiding places during their spawning migrations (see Supporting Information S1). They have to swim occasionally to the surface to inflate their swim bladder, which makes this species vulnerable to collisions with passing vessels or ship propeller strikes (Spierts 2016). In 2012 and 2015, three of a total of 87 experimentally released juvenile European sturgeons were found dead along the Rhine river banks, showing sharp cuts, most probably caused by ship propeller strikes (Brevé et al. 2019). Unfortunately, such events are likely to increase during dry summer months as ships and fish are forced into a narrowed shipping lane. Because of climate change Alpine glaciers are melting and will disappear, which causes the Rhine river discharge to also reduce (Stahl et al. 2022). Moreover, river bed erosion and continuous dredging and nourishment (augmentation) of bed sediment within the river's deepest parts to maintain the shipping lane (Frings 2015) are known to reduce the availability and quality of spawning and nursery sites for the Atlantic sturgeon ( *A. oxyrinchus* ), sister species of European sturgeon (Balazik et al. 2020; Barber 2017; Nellis et al. 2007). In the Rhine main river stem, especially between the towns of Nijmegen and Cologne, shipping intensity causes an almost permanent disturbance (Staas 2017; van de Ven 2021). Passing ships create waves and water displacement, which may affect egg and yolk‐sac larvae survival at the spawning sites (Gabel, Lorenz, and Stoll 2017; Spierts 2016). Therefore, inland navigation and river bed maintenance could seriously impair the quality of the Rhine for the European sturgeon's youngest life phases.

### 4.4 Opportunities to Improve Key Sturgeon Habitats and Implement Conservation Measures

This study determined the availability and quality of spawning and nursery habitats for sturgeon in the Rhine. The results may also indicate opportunities for active habitat restoration and protection. As for the dams, the discharge sluices of the Haringvliet dam that block fish migration between the former Rhine estuary and the North Sea, only a change in the management of the sluices would allow for a permanent estuary gradient and migration corridor. However, as this would have drastic consequences for the freshwater supply in the region, it is not considered a realistic scenario in the near future. Considering the negative effects of inland navigation in the Lower Rhine on sturgeon and their key habitats at least three adaptations is considered promising, as suggested by (Spierts 2016; Staas 2017; van de Ven 2021). One adaptation would be to divide the main channel into two parallel channels, as has been done in the Room for the River pilot ‘longitudinal training wall’. Over a distance of 10 km, such structures significantly reduce the impact of passing vessels (Collas et al. 2018). Other adaptations would be to adjust the shipping lane to avoid key spawning sites and reduce vessel speed and the number of revolutions per minute of their propellers. This latter would reduce harmful water displacement and the likelihood of propeller strikes.

### 4.5 Implications for Conservation

The insights from the current study on spawning and nursery habitat requirements of the European sturgeon can be used to assess the current suitability of the river Rhine for the species' reintroduction and to identify opportunities for habitat restoration and protection for various life stages. The outcomes thus play an essential role in the conservation of the species. In addition, the modelling approach developed to map spawning and nursery habitats for the Rhine could be applied to other northwestern European rivers. This broader application would allow intercomparison and support decisions about which rivers are best suited for future reintroductions of the critically endangered European sturgeon.

## Author Contributions

**Niels W. P. Brevé:** conceptualization, methodology, validation, formal analysis, data curation, writing–original draft preparation, investigation, visualization, project administration. **Debora A. J. van Dieren:** methodology, validation, resources, data curation, formal analysis. **Marc Weeber:** methodology, validation, formal analysis, project administration, writing–review and editing. **Erik Mosselman:** methodology, formal analysis, writing–review and editing. **Leopold A. J. Nagelkerke** and **AlberTinka J. Murk:** writing–review and editing. **Anthonie D. Buijse:** funding acquisition, conceptualisation, methodology, validation, writing–review and editing, supervision.

## Conflicts of Interest

The authors declare no conflicts of interest.

## Supporting information


**Data S1.** HSI‐parameter‐setting for modelling and GIS‐based mapping of European sturgeon reproductive habitat, including descriptions per life stage and literature references.

**Data S2** . Data modeling approach in two parts.

## Data Availability

The data that support the findings of this study are available online; see Table 1.
