# Supplementary material for: Assessing Potential Spawning and Nursery Habitat Availability in the River Rhine for the Critically Endangered European Sturgeon
Source: Aquat Conserv. 2024 Nov 25;34(11):e70016. doi: 10.1002/aqc.70016 (PMC11588678; doi:10.1002/aqc.70016)
Supplement: Supplementary file 2 — Data S2 . Data modeling approach in two parts. [file AQC-34-e70016-s002.docx]

SUPPLEMENTARY INFORMATION

**Modelling reproductive habitat availability of the critically endangered European sturgeon in a highly modified European river**

APPENDIX A

Please note, Appendix A is a separate Excel file that has the references of a systematic literature review on the European sturgeons’ reproductive habitat parameters.

**(In the paper, see paragraph 2.3)**

APPENDIX B

**Part 1. (Hydrodynamic) translation
For transforming 1D model results and measurements in 2D information**

**(In the paper, see paragraph 2.4)**

**Modelling flow velocity and water depth**

**Mapping a 1D model result to 2D distributions**

*Water depth*

*Flow velocity*

*Sediment*

**Part 2. Deriving the Habitat Suitability Index**

**(In the paper, see paragraph 2.5)**

**Calculation approach**

**YAML configuration**

**Part 1. (Hydrodynamic) translation used for transforming 1D model results and measurements in 2D information (see paragraph 2.4)**

**Modelling flow velocity and water depth**

To map the 2D distributions of flow velocity and water depth for the study area (until the tidal zone) we combined data of the riverbed topography with a 1D SOBEK model (<https://www.deltares.nl/en/software-and-data/products/sobek-suite> ), composed of: Rhine-Meuse mouth *sobek-rmm-vzm-j15_5-v4,* Rhine branches (Rijntakken) *sobek-rijn-j22_6-v1a1_rwsos*, and for the German part of the Rhine *sobek-rijn-j17_5-v2-rwsos_merge*.

**Mapping a 1D model result to 2D distributions**

Initially, the average, seasonal river discharge for spawning sites (May-July), and for nursery sites (June-March), however after concluding that these did not differ significantly only one average, seasonal river discharge distribution was used for spawning and nursery sites (June-Aug) . To include discharge variability percentiles were calculated over yearly, average river discharges, to represent dry (p10), average (p50) and wet years (p90).

The 1D SOBEK model outputs the water level and river discharge at observation points, which occur at every river kilometer. The SOBEK model **cross sections** occur every 500 m in the Dutch part and every 200 m in the German part. These cross sections are based on the relationship between water level and river discharge, and are only applicable to the river part located within the summer dikes (Fig. 5).

**Figure S1:** Example of a SOBEK crossection. These crosssections describe the dimensions of the river and occur every 500 m in the Dutch part and every 200m in the German part.


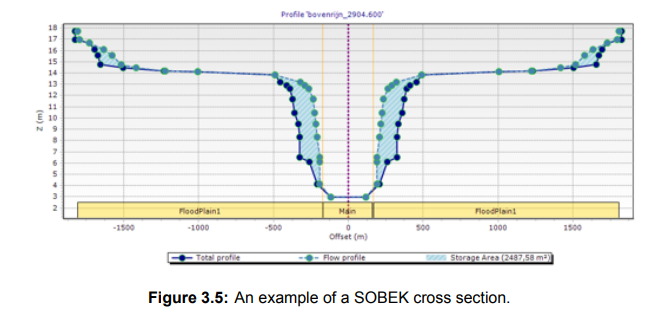


*Water depth*

The water levels from the merged 1D SOBEK model are combined with bed topography data, in order to map water depths (fig. 3). The bed topography data has a spatial resolution of 1x1m^2^ and is provided by RIJKSWATERSTAAT and WSV. The water depth *d* of every grid cell *k* was derived from the bottom level *z* at the grid cell and the water level *h* from the nearest observation point, using the formula:

d_k = h – z_k.

Areas that were not connected to the main river were removed, as those areas are not of interest for our study as they cannot be accessed by sturgeons. We did not consider extreme situations in which these area would become accessible due to flooding.

**Figure S2:** Explanation of a SOBEK 1D model schematization based on a river stretch.The SOBEK output is given at the observation points. The water level is assumed constant in the area around that point (orange area). The smoothness is assumed constant in the area around a SOBEK cross section (blue area). The blue and orange area are determined by nearest neighbour. These results are translated to a 1x1 m^2^ resolution grid cells based on the bed topography supplied. The depth and flow velocity maps have 1x1m^2^ grid cells.


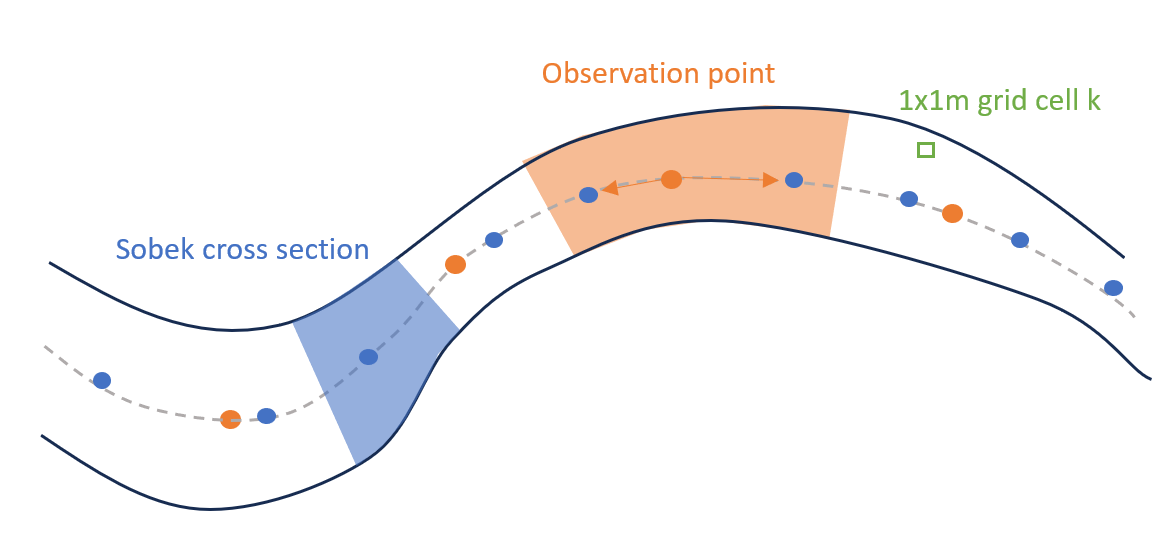


*Flow velocity*

The flow velocity map, based upon the 1D model, is partially created by calibration. This was done using the Chezy formula for every SOBEK cross-section, and applying the Chezy formula to the map that has the water depths.

Cross sections were divided into 150 vertical bins with equal width $\Delta B$. The depth of each bin was calculated, using the cross section profile and the water level of the observation point closest to the cross section.

Using the Chézy formula

**Equation S1**


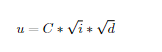


And the discharge relation

**Equation S2**


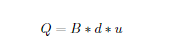


Where u is the flow velocity [m/s], C is the Chezy coefficient [$\sqrt{m}/s]$, i is the hydraulic gradient [m/m] and d is the depth [m], Q is the discharge [$m^{3}/s$], and $B$ is the river width [m].

These formulas are combined and applied per bin j:

**Equation S3**


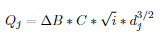


Where $Q_{j}$ is the discharge of a bin [$m^{3}/s$], $\Delta B$ is the bin width [m], C is the Chezy coefficient [$\sqrt{m}/s]$, i is the hydraulic gradient [m/m] and $d_{j}$ is the depth of a bin [m].

The total discharge is equal to the sum of the discharges per bin. This leads to

**Equation S4**


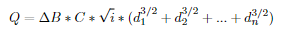


The smoothness factor $C\sqrt{i}$ of a cross section can then be determined using

**Equation S5**


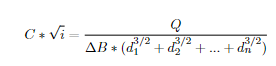


The smoothness factor is assumed to be constant over a cross section. However, the Chezy constant *C* and hydraulic gradient *i* values can vary over a cross section. For example, vegetation reduces the smoothness locally. Additionally, normal flow as assumed in the Chézy formula is not present in contracting and expanding flows, for example in groyne fields. Third of all, the official definition of the Chézy formula does not contain the water depth *d*, but the hydraulic radius R. These two are the same in wide channels, which holds for the study.

The same smoothness factor is used for the entire area surrounding the SOBEK cross section. To determine the flow velocity in every grid cell k, the Chézy formula is applied:

**Equation S6**

$u_{k}=\alpha*\left( C*\sqrt{i} \right)*\sqrt{d_{k}}$

Where $u_{k}$ is the flow velocity in grid cell k $[ m/s$], C is the Chezy coefficient [$\sqrt{m}/s]$, i is the hydraulic gradient [m/m] and $d_{k}$ is the depth in grid cell k [m].

In this formula, a calibration factor $\alpha$ is inserted. This calibration factor was derived by a comparison to a 2D hydrodynamic model dflowfm2d-rijn-j22_6-v1a (Kosters & Visser, 2022). The calibration values follow from a comparison of the flow velocity values in a 10x10 km^2^ test area, where flow velocities from the 2D model and flow velocities from our 1D-to-map approach are compared. This showed that the effect of vegetation and groyne fields were not captured by our method, as these factors decrease the flow velocity. With calibration the smoothness factor in the shallowest areas was lowered to correct for these effects.The calibration increases the flow velocity in deeper areas as the flow velocity was underestimated there. Calibration is done for every area around a cross section (blue area in figure S2). Of such area, the shallowest 0-10% is calibrated by a factor 0.53, the 10-20% shallowest area was calibrated by a factor 1.06, and so on. Table S1 shows the calibration values.

**Table S1:** The calibration factor alpha per depth interval.

| Interval from % | Interval to % | Calibration factor $\alpha$ |
| --- | --- | --- |
| 0 | 10 | 0.53 |
| 10 | 20 | 1.06 |
| 20 | 30 | 1.30 |
| 30 | 40 | 1.32 |
| 40 | 50 | 1.28 |
| 50 | 60 | 1.24 |
| 60 | 70 | 1.24 |
| 70 | 80 | 1.27 |
| 80 | 90 | 1.24 |
| 90 | 100 | 1.17 |

By using equation S6, the flow velocities outside the main channel, such as in lakes and harbours, are overestimated. All flow velocities in those areas remain below 0.5 m/s. Therefore, the flow velocities outside the main channel are set to 0.1 m/s, such that the lakes and harbours will fall in the low flow velocity category 0-0.5 m/s.

*Sediment*

The sediment maps are generated based on measurements of the mean sediment grain size (D50). From the D50 measurements in Germany and the Rijntakken area, cross sections with multiple measurements are identified. Here the sediment pattern is heterogeneous , with sediment sizes varying considerably within individual cross sections. . In the Rhine-Meuse mouth area, the original spatial data was unavailable, and nearly all D50 values in that region fell within the sediment size category of 0.075-2 mm. Therefore, a D50 value of 1 mm is applied to the entire Rhine-Meuse mouth area to categorize it within the 0.075-2 mm sediment size category.

For the spawning stage, the maximum and median D50 values are considered for each cross section. Maximum D50 values are used since larger sediment is required for spawning, while median D50 values are included to illustrate the influence of this heterogeneity. For the nursery life stage, minimum and median D50 values are taken for each cross section. Minimum D50 values are chosen because smaller sediment is required for the nursery life stage, and median D50 values highlight the effects of the sediment's heterogeneity.

These values are then spatially distributed across the entire map using a nearest neighbor method.

**Part 2. Deriving the habitat suitability index (see paragraph 2.5)**

**Calculation approach**

Based on the flow velocity, water depth and sediment maps created in the previous steps a Habitat Suitability Index (HSI) is derived for sturgeon nursery and spawning habitat. D-Eco Impact (<https://github.com/Deltares/D-EcoImpact> ; <https://www.deltares.nl/en/software-and-data/products/d-eco-impact> ), a python-based spatial ecological assessment module, is used to derive the HSI per map and variable. Its calculation determines the effect of the variation in discharge and sediment size on suitable spawning and nursery habitat. All variables where assessed based on a stepped classification of the HSI. The overall HSI for spawning and nursery habitat is derived based on the minimum feasibility of the variable specific HSI values, using the following formula:

**Equation S7**

HSI_overall = MIN( HSI_flow_velocity, HSI_water_depth, HIS_sediment_size)

For each individual variable and the overall result the area associated with a HSI score is calculated. This provides an indication of band width in which our model results should be considered, describing the uncertainty in discharge scenarios and the effect of sediment size heterogeneity in the river. To determine the most limiting variable for the overall HSI score of suitable spawning or nursery the contribution of each variable to the overall score is investigated, providing a spatial overview of where which variable is limiting to the overall result.

**YAML configuration**

Due to computational limits the input of each variable was aggregated from a 1x1m^2^ to a 2x2m^2^ raster grid. Each raster tile was assessed with D-Eco Impact v0.3.0 based on the code shown in S1.

**Code S1** To computate the resulting HSI values and limitations the following YAML configuration was used with D-Eco Impact to dynamically assess the each raster tile containing the variables water depth, flow velocity and sediment size.

---------------------------------------------------------------------------------------------------------------

**version**: 0.1.0

**input-data**:

**- dataset**:

**filename**: {input_netcdf}

**variable_mapping**:

**waterdepth_mNAP**: "INPUT_waterdepth_mNAP"

**velocity_ms**: "INPUT_velocity_ms"

**sedimentsizemax_mm**: "INPUT_sedimentsizemax_mm"

**sedimentsizemed_mm**: "INPUT_sedimentsizemed_mm"

**sedimentsizemin_mm**: "INPUT_sedimentsizemin_mm"

**rules**:

#Fertelized eggs & Yolk-sac larvae

**- step_function_rule**:

**name**: Suitability waterdepth eggs

**description**: Suitability waterdepth eggs

**limit_response_table**:

- [ limit , response]

- [ 0.0 , 0.25]

- [ 2.0 , 1.00]

- [ 4.0 , 0.75]

- [ 6.0 , 0.50]

- [ 999 , 0.00]

**input_variable**: INPUT_waterdepth_mNAP

**output_variable**: suit_waterdepth_eggs

**- step_function_rule**:

**name**: Suitability flow velocity eggs

**description**: Suitability flow velocity eggs

**limit_response_table**:

- [ limit , response]

- [ 0.0 , 0.50]

- [ 0.5 , 1.00]

- [ 1.0 , 0.75]

- [ 1.5 , 0.50]

- [ 2.0 , 0.00]

- [ 999 , 0.00]

**input_variable**: INPUT_velocity_ms

**output_variable**: suit_velocity_eggs

**- step_function_rule**:

**name**: Suitability sediment median grain size eggs

**description**: Suitability sediment medium grain size eggs

**limit_response_table**:

- [ limit , response]

- [ 0.0 , 0.00]

- [ 0.075 , 0.00]

- [ 2.0 , 0.00]

- [ 16.0 , 1.00]

- [ 31.5 , 0.75]

- [ 999.0 , 0.00]

**input_variable**: INPUT_sedimentsizemed_mm

**output_variable**: suit_sedimentmed_eggs

**- step_function_rule**:

**name**: Suitability sediment maximum grain size eggs

**description**: Suitability sediment maximum grain size eggs

**limit_response_table**:

- [ limit , response]

- [ 0.0 , 0.00]

- [ 0.075 , 0.00]

- [ 2.0 , 0.00]

- [ 16.0 , 1.00]

- [ 31.5 , 0.75]

- [ 999.0 , 0.00]

**input_variable**: INPUT_sedimentsizemax_mm

**output_variable**: suit_sedimentmax_eggs

**- combine_results_rule**:

**name**: HSI eggs med grainsize

**description**: Minimum habitat suitability for eggs based on flow velocity,waterdepth and median sediment

**operation**: min

**input_variables**: ["suit_waterdepth_eggs","suit_velocity_eggs","suit_sedimentmed_eggs"]

**output_variable**: HSI_eggs_sedimentmed

**- combine_results_rule**:

**name**: HSI eggs max grainsize

**description**: Minimum habitat suitability for eggs based on flow velocity, waterdepth and maximum sediment

**operation**: min

**input_variables**: ["suit_waterdepth_eggs","suit_velocity_eggs","suit_sedimentmax_eggs"]

**output_variable**: HSI_eggs_sedimentmax

**- classification_rule**:

**name**: Most limiting variable HSI_eggs_sedimentmed

**description**: Most limiting variable HSI_eggs_sedimentmed

**criteria_table**:

- ["output", "HSI_eggs_sedimentmed", "suit_waterdepth_eggs","suit_velocity_eggs","suit_sedimentmed_eggs"]

- [ 1 , "0.0" , "<0.01" , "-" , "-"] # Limit waterdepth (0.0)

- [ 2 , "0.0" , "-" , "<0.01" , "-"] # Limit flow velocity (0.0)

- [ 3 , "0.0" , "-" , "-" , "<0.01"] # Limit sedimentmed (0.0)

- [ 4 , "0.25" , "<0.26" , "-" , "-"] # Limit waterdepth (0.25)

- [ 5 , "0.25" , "-" , "<0.26" , "-"] # Limit flow velocity (0.25)

- [ 6 , "0.25" , "-" , "-" , "<0.26"] # Limit sedimentmed (0.25)

- [ 7 , "0.5" , "<0.51" , "-" , "-"] # Limit waterdepth (0.5)

- [ 8 , "0.5" , "-" , "<0.51" , "-"] # Limit flow velocity (0.5)

- [ 9 , "0.5" , "-" , "-" , "<0.51"] # Limit sedimentmed (0.5)

- [ 10 , "0.75" , "<0.76" , "-" , "-"] # Limit waterdepth (0.75)

- [ 11 , "0.75" , "-" , "<0.76" , "-"] # Limit flow velocity (0.75)

- [ 12 , "0.75" , "-" , "-" , "<0.76"] # Limit sedimentmed (0.75)

- [ 13 , "1.0" , "-" , "-" , "-"] # No or no clear limit

**input_variables**: [ "HSI_eggs_sedimentmed", "suit_waterdepth_eggs","suit_velocity_eggs","suit_sedimentmed_eggs"]

**output_variable**: limit_HSI_eggs_sedimentmed

**- classification_rule**:

**name**: Most limiting variable HSI_eggs_sedimentmax

**description**: Most limiting variable HSI_eggs_sedimentmax

**criteria_table**:

- ["output", "HSI_eggs_sedimentmax", "suit_waterdepth_eggs","suit_velocity_eggs","suit_sedimentmax_eggs"]

- [ 1 , "0.0" , "<0.01" , "-" , "-"] # Limit waterdepth (0.0)

- [ 2 , "0.0" , "-" , "<0.01" , "-"] # Limit flow velocity (0.0)

- [ 3 , "0.0" , "-" , "-" , "<0.01"] # Limit sedimentmax (0.0)

- [ 4 , "0.25" , "<0.26" , "-" , "-"] # Limit waterdepth (0.25)

- [ 5 , "0.25" , "-" , "<0.26" , "-"] # Limit flow velocity (0.25)

- [ 6 , "0.25" , "-" , "-" , "<0.26"] # Limit sedimentmax (0.25)

- [ 7 , "0.5" , "<0.51" , "-" , "-"] # Limit waterdepth (0.5)

- [ 8 , "0.5" , "-" , "<0.51" , "-"] # Limit flow velocity (0.5)

- [ 9 , "0.5" , "-" , "-" , "<0.51"] # Limit sedimentmax (0.5)

- [ 10 , "0.75" , "<0.76" , "-" , "-"] # Limit waterdepth (0.75)

- [ 11 , "0.75" , "-" , "<0.76" , "-"] # Limit flow velocity (0.75)

- [ 12 , "0.75" , "-" , "-" , "<0.76"] # Limit sedimentmax (0.75)

- [ 13 , "1.0" , "-" , "-" , "-"] # No or no clear limit

**input_variables**: [ "HSI_eggs_sedimentmax", "suit_waterdepth_eggs","suit_velocity_eggs","suit_sedimentmax_eggs"]

**output_variable**: limit_HSI_eggs_sedimentmax

#Larvae (<ca. 50 mm) and juveniles 0+

**- step_function_rule**:

**name**: Suitability waterdepth juvenile

**description**: Suitability waterdepth juvenile

**limit_response_table**:

- [ limit , response]

- [ 0.0 , 0.75]

- [ 2.0 , 1.00]

- [ 4.0 , 1.00]

- [ 6.0 , 0.75]

- [ 999 , 0.00]

**input_variable**: INPUT_waterdepth_mNAP

**output_variable**: suit_waterdepth_juvenile

**- step_function_rule**:

**name**: Suitability flow velocity juvenile

**description**: Suitability flow velocity juvenile

**limit_response_table**:

- [ limit , response]

- [ 0.0 , 1.00]

- [ 0.5 , 0.50]

- [ 1.0 , 0.25]

- [ 1.5 , 0.00]

- [ 2.0 , 0.00]

- [ 999 , 0.00]

**input_variable**: INPUT_velocity_ms

**output_variable**: suit_velocity_juvenile

**- step_function_rule**:

**name**: Suitability sediment median grain size juvenile

**description**: Suitability sediment median grain size juvenile

**limit_response_table**:

- [ limit , response]

- [ 0.0 , 1.00]

- [ 0.075 , 0.75]

- [ 2.0 , 0.25]

- [ 16.0 , 0.0 ]

- [ 31.5 , 0.0 ]

- [ 999.0 , 0.0 ]

**input_variable**: INPUT_sedimentsizemed_mm

**output_variable**: suit_sedimentmed_juvenile

**- step_function_rule**:

**name**: Suitability sediment minimum grain size juvenile

**description**: Suitability sediment minimum grain size juvenile

**limit_response_table**:

- [ limit , response]

- [ 0.0 , 1.00]

- [ 0.075 , 0.75]

- [ 2.0 , 0.25]

- [ 16.0 , 0.0 ]

- [ 31.5 , 0.0 ]

- [ 999.0 , 0.0 ]

**input_variable**: INPUT_sedimentsizemin_mm

**output_variable**: suit_sedimentmin_juvenile

**- combine_results_rule**:

**name**: HSI juvenile

**description**: Minimum habitat suitability for juvenile based on flow velocity, waterdepth and median sediment

**operation**: min

**input_variables**: ["suit_waterdepth_juvenile","suit_velocity_juvenile","suit_sedimentmed_juvenile"]

**output_variable**: HSI_juvenile_sedimentmed

**- combine_results_rule**:

**name**: HSI juvenile

**description**: Minimum habitat suitability for juvenile based on flow velocity, waterdepth and minimum sediment

**operation**: min

**input_variables**: ["suit_waterdepth_juvenile","suit_velocity_juvenile","suit_sedimentmin_juvenile"]

**output_variable**: HSI_juvenile_sedimentmin

**- classification_rule**:

**name**: Most limiting variable HSI_juvenile_sedimentmed

**description**: Most limiting variable HSI_juvenile_sedimentmed

**criteria_table**:

- ["output", "HSI_juvenile_sedimentmed", "suit_waterdepth_juvenile","suit_velocity_juvenile","suit_sedimentmed_juvenile"]

- [ 1 , "0.0" , "<0.01" , "-" , "-"] # Limit waterdepth (0.0)

- [ 2 , "0.0" , "-" , "<0.01" , "-"] # Limit flow velocity (0.0)

- [ 3 , "0.0" , "-" , "-" , "<0.01"] # Limit sedimentmed (0.0)

- [ 4 , "0.25" , "<0.26" , "-" , "-"] # Limit waterdepth (0.25)

- [ 5 , "0.25" , "-" , "<0.26" , "-"] # Limit flow velocity (0.25)

- [ 6 , "0.25" , "-" , "-" , "<0.26"] # Limit sedimentmed (0.25)

- [ 7 , "0.5" , "<0.51" , "-" , "-"] # Limit waterdepth (0.5)

- [ 8 , "0.5" , "-" , "<0.51" , "-"] # Limit flow velocity (0.5)

- [ 9 , "0.5" , "-" , "-" , "<0.51"] # Limit sedimentmed (0.5)

- [ 10 , "0.75" , "<0.76" , "-" , "-"] # Limit waterdepth (0.75)

- [ 11 , "0.75" , "-" , "<0.76" , "-"] # Limit flow velocity (0.75)

- [ 12 , "0.75" , "-" , "-" , "<0.76"] # Limit sedimentmed (0.75)

- [ 13 , "1.0" , "-" , "-" , "-"] # No or no clear limit

**input_variables**: [ "HSI_juvenile_sedimentmed", "suit_waterdepth_juvenile","suit_velocity_juvenile","suit_sedimentmed_juvenile"]

**output_variable**: limit_HSI_juvenile_sedimentmed

**- classification_rule**:

**name**: Most limiting variable HSI_juvenile_sedimentmin

**description**: Most limiting variable HSI_juvenile_sedimentmin

**criteria_table**:

- ["output", "HSI_juvenile_sedimentmin", "suit_waterdepth_juvenile","suit_velocity_juvenile","suit_sedimentmin_juvenile"]

- [ 1 , "0.0" , "<0.01" , "-" , "-"] # Limit waterdepth (0.0)

- [ 2 , "0.0" , "-" , "<0.01" , "-"] # Limit flow velocity (0.0)

- [ 3 , "0.0" , "-" , "-" , "<0.01"] # Limit sedimentmin (0.0)

- [ 4 , "0.25" , "<0.26" , "-" , "-"] # Limit waterdepth (0.25)

- [ 5 , "0.25" , "-" , "<0.26" , "-"] # Limit flow velocity (0.25)

- [ 6 , "0.25" , "-" , "-" , "<0.26"] # Limit sedimentmin (0.25)

- [ 7 , "0.5" , "<0.51" , "-" , "-"] # Limit waterdepth (0.5)

- [ 8 , "0.5" , "-" , "<0.51" , "-"] # Limit flow velocity (0.5)

- [ 9 , "0.5" , "-" , "-" , "<0.51"] # Limit sedimentmin (0.5)

- [ 10 , "0.75" , "<0.76" , "-" , "-"] # Limit waterdepth (0.75)

- [ 11 , "0.75" , "-" , "<0.76" , "-"] # Limit flow velocity (0.75)

- [ 12 , "0.75" , "-" , "-" , "<0.76"] # Limit sedimentmin (0.75)

- [ 13 , "1.0" , "-" , "-" , "-"] # No or no clear limit

**input_variables**: [ "HSI_juvenile_sedimentmin", "suit_waterdepth_juvenile","suit_velocity_juvenile","suit_sedimentmin_juvenile"]

**output_variable**: limit_HSI_juvenile_sedimentmin

**output-data**:

**filename**: {output_netcdf}
